# Comparative Study Between Minimally Invasive Plate Osteosynthesis and Distal Tip Locking Tibial Nailing in the Treatment of Lower Third Tibial Shaft Fractures

**DOI:** 10.7759/cureus.97136

**Published:** 2025-11-18

**Authors:** Abdelrahman Sayed, Ahmed Naeem, Ali Soffar, Ahmed Elkohail, Mamdouh Elbannan, Mohammad Abdalla, Abd Elhamed Attallah

**Affiliations:** 1 Trauma and Orthopaedics, Cardiff and Vale Health Board, Cardiff, GBR; 2 Trauma and Orthopaedics, Al-Azhar University Hospitals, Cairo, EGY; 3 Trauma and Orthopaedics, King's College NHS Foundation Trust, London, GBR; 4 Trauma and Orthopaedics, Swansea Bay University, Swansea, GBR

**Keywords:** distal locked plate, distal tibia, fracture, interlocking nail (iln), internal fixation, mippo

## Abstract

Background: Distal third tibial shaft fractures are challenging due to limited soft tissue coverage and poor vascularity. We compared minimally invasive plate osteosynthesis (MIPO) with distal tip-locking intramedullary nailing.

Methods: A prospective two-center cohort of 40 adults with extra-articular distal third tibial fractures was allocated to MIPO (n = 20) or intramedullary nailing (IMN) (n = 20). Primary outcomes were time to union and six-month functional results (AOFAS). Secondary outcomes included infections, malalignment, and secondary procedures.

Results: Mean age was similar (MIPO 35.22 ± 5.53 years; IMN 35.52 ± 5.90). Sex distribution was as follows: MIPO 15 (75%) men and five (25%) women; IMN 14 (70%) men and six (30%) women. Union was faster with IMN (14 ± 2.5 weeks) than with MIPO (17 ± 4 weeks). AOFAS at six months showed that in the MIPO group, 12 (60%) were excellent, six (30%) were good, and two (10%) were fair. In the IMN group, 14 (70%) were excellent, four (20%) were good, and two (10%) were fair. Superficial infection occurred in four (20%) in the MIPO group versus three (15%) in the IMN group; no infection occurred in 16 (80%) in the MIPO group versus 17 (85%) in the IMN group. Malalignment occurred in one (5%) in the MIPO group versus three (15%) in the IMN group. Secondary procedures in the MIPO group included one (5%) revision and three (15%) debridements, with 16 (80%) requiring none. In the IMN group, two (10%) required dynamization, and 18 (90%) required no additional procedure.

Conclusions: For extra-articular distal third tibial fractures, IMN achieved faster union and a higher proportion of excellent AOFAS outcomes, with similar low rates of complications, whereas MIPO showed a lower malalignment rate. Both techniques are viable, with IMN favored when earlier union is prioritized.

## Introduction

Mechanical overload can cause a fracture, resulting in serious physiological repercussions. Choosing the best treatment option for a particular fracture requires a thorough understanding of the biological and mechanical elements of fracture repair [1]. Tibial fractures are the most common long-bone fractures; in younger individuals, sports-related injuries are the most prevalent cause, whereas in the elderly, simple falls predominate [2]. The fracture pattern of tibial injuries is typically simple and associated with less severe soft-tissue damage compared with open tibial fractures, while complex configurations are more commonly observed in older, less physically active individuals with osteoporotic bone [3].

The five primary causes of tibial fractures are sports injuries, falls, direct blows or assaults, gunshot wounds, and motor vehicle accidents. Falls can be categorized as simple falls (from standing height or down stairs or slopes) and falls from a height (FFH) [4-7]. These injuries typically require hospitalization and often necessitate surgical intervention. The tibial diaphysis is the most common site, and approximately 80% of these fractures are associated with fibular fractures [8].

Fractures of the distal tibial shaft account for about 37.8% of all tibial injuries and affect individuals of all ages. Management in skeletally mature patients without articular involvement is challenging due to inadequate vascular supply, subcutaneous location, and proximity to the ankle. The lack of muscle coverage, injury complexity, and poor vascularity make these fractures difficult to treat. Traditionally, distal tibial fractures were managed with open reduction and internal fixation (ORIF); however, due to the high incidence of complications, alternative fixation methods such as intramedullary nailing (IMN) and minimally invasive plate osteosynthesis (MIPO) have become increasingly popular [9].

Some authors recommend external fixation combined with limited ORIF, citing minimal soft-tissue complications and favorable functional outcomes. The advancement of biological fixation techniques and locking plates has led to increased use of MIPO, which is considered reliable and safe, while IMN allows minimally invasive, stable, and dynamic fixation and has been extensively used with good outcomes. Each technique has distinct advantages and disadvantages. Nonoperative management may lead to joint stiffness in up to 40% of cases and shortening or rotational malunion in more than 30% [10].

## Materials and methods

Study design and setting

This was a prospective comparative cohort study conducted from June 2022 to June 2024 at Al-Zahraa Hospital and Aboukir General Hospital. The study included a total of 40 patients with extra-articular distal third tibial fractures managed either with an expert tibial nail inserted through a transpatellar approach or with a distal tibial locked plate inserted using the MIPO technique. Ethical approval was obtained from the Research Ethics Committee of the Faculty of Medicine for Girls, Cairo, Al-Azhar University (FMG-IRB; approval 2022051353).

The inclusion criteria were all skeletally mature patients and closed or open Gustilo I fractures. The exclusion criteria were skeletally immature patients, intra-articular fractures, fractures associated with neurovascular injuries, open fractures with Gustilo level II or above, pathological fractures, and polytrauma patients. All patients underwent preoperative assessment and optimization of comorbidities. Evaluation and assessment were standardized for all participants [11,12].

Outcome parameters encompassed intraoperative and postoperative measures. Intraoperatively, we recorded the duration of surgery (minutes), estimated blood loss (based on soaked gauze count; one fully soaked gauze ≈ 70 mL), length of the surgical incision, and any vascular or neurological injury. Postoperatively, we assessed the rate of infection, ankle and knee range of motion, scar or keloid formation, time to union, time to full weight-bearing, and the radiological union score (RUST) [13].

Allocation to MIPO or IMN was nonrandom and determined by the treating surgeon’s clinical judgment, considering fracture morphology (AO/OTA classification), soft-tissue status, patient comorbidities, and implant availability. No alternating schedules or concealed randomization were used.

Surgical techniques

Plate Fixation (MIPO Group)

All procedures were performed in the supine position on a radiolucent table under tourniquet control. The fibula was fixed if the fracture was located within 10 cm of the fibular tip. All cases were treated using the MIPO technique, which involved two small incisions over the distal and proximal ends of the plate, with the remaining screws inserted percutaneously.

Intramedullary Nailing (IMN Group)

Patients in the nailing group were positioned supine on a radiolucent table under tourniquet control. The procedure was standardized and performed through a transpatellar approach.

Postoperative management

Postoperative radiographs of the entire leg were taken in both anteroposterior and lateral views. Neurovascular status was assessed, and intravenous broad-spectrum antibiotics were administered for two days. Low-molecular-weight heparin (LMWH) was given every 24 hours postoperatively until mobilization as prophylaxis against deep vein thrombosis (DVT) and pulmonary embolism (PE). Patients were discharged on the third postoperative day with prescriptions for one week of oral antibiotics, anti-edematous medications, and analgesics. They were advised to perform ankle and knee range-of-motion and quadriceps strengthening exercises, with restricted weight-bearing initially.

The follow-up protocol included reviews at two, four, six, and twelve weeks, and at six months. At two weeks, the wound was inspected, sutures were removed, infection was assessed, and partial weight-bearing with crutches was advised. At four weeks, radiographs were obtained to evaluate fixation stability, wound healing, and skin condition. At six weeks, X-rays assessed callus formation, ankle and knee motion were examined, and physiotherapy was initiated if stiffness was present. At 12 weeks, X-rays were taken to check for union or fixation failure. At six months, a final radiographic assessment confirmed complete union, full weight-bearing was permitted, and the AOFAS score was recorded [14].

Evaluation and assessment methods

Clinical and Radiographic Union

Clinical union was defined as the absence of focal tenderness at the fracture site, painless full weight-bearing, and a normal gait. Radiographic union was graded using the Radiographic Union Score for Tibial fractures (RUST): 1 = visible fracture line without callus, 2 = callus with a visible fracture line, and 3 = bridging callus with no fracture line; totals range from 4 (not healed) to 12 (fully healed) [13,15,16].

Alignment Assessment

Malalignment was defined as sagittal angulation (antecurvatum/recurvatum) >5°, coronal angulation (varus/valgus) >5°, or rotational malalignment ≥15°. Rotational alignment was assessed with the patient supine and both patellae facing anteriorly; the angle between the lateral border of the foot and the examination table was measured and compared bilaterally.

Functional Outcome

Functional status at six months was measured using the American Orthopaedic Foot and Ankle Society (AOFAS) ankle-hindfoot score: ≥90 excellent, 75-89 good, 50-74 fair, and <50 poor.

Statistical analysis

All data were collected, tabulated, and analyzed using IBM SPSS Statistics for Windows, Version 26 (Released 2019; IBM Corp., Armonk, New York). Qualitative variables were expressed as numbers and percentages, while quantitative variables were expressed as mean ± standard deviation (SD), median, and range. Statistical comparisons were two-tailed, with p ≤ 0.05 considered significant and p < 0.001 highly significant. The chi-square (χ²) test was used for comparing categorical data, and the independent t-test was used for comparing continuous data between groups.

## Results

Baseline characteristics

In the MIPO group (n = 20), the mean age was 35.22 ± 5.53 years (range 20-50), and in the IMN group (n = 20), the mean age was 35.52 ± 5.9 years (range 20-50). In the MIPO group, 15 (75%) were men and 5 (25%) were women; in the IMN group, 14 (70%) were men and 6 (30%) were women. Comparison of demographic data between the two groups showed no significant differences with respect to sex or age. In the MIPO group, AO 43A1.1 was 2 (10%), 43A1.2 was 6 (30%), 43A1.3 was 2 (10%), 43A2.1 was 2 (10%), 43A2.2 was 4 (20%), and 43A2.3 was 4 (20%). In the IMN group, AO 43A1.1 was 10 (50%), 43A1.2 was 4 (20%), 43A1.3 was 4 (20%), 43A2.1 was 0 (0%), 43A2.2 was 0 (0%), and 43A2.3 was 2 (10%). There was a highly significant difference between the two groups in AO fracture classification (χ² = 6, p < 0.001). A statistically significant difference was also found regarding fibular fixation (χ² = 4, p = 0.04): in the MIPO group, 2 (10%) fibulae were intact, 10 (50%) were fixed, and 8 (40%) were not fixed, compared with 3 (15%), 7 (35%), and 10 (50%) in the IMN group, respectively. Intraoperative blood loss was significantly lower in the MIPO group (91.1 ± 25.2 mL; median 100, range 70-120 mL) than in the IMN group (222.1 ± 70.1 mL; median 250, range 150-300 mL; t = 16, p < 0.001) (Table 1).

**Table 1:** Comparative analysis among both groups as regard baseline characteristics A p-value above 0.05 was considered nonsignificant; a p-value under 0.05 was considered significant. MIPO: minimally invasive plate osteosynthesis, IMN: intramedullary nailing, SD: standard deviation, AO: Arbeitsgemeinschaft für Osteosynthesefragen, X²: chi-square, T: two-sample independent t-test.

Variables	MIPO (n = 20)	IMN (n = 20)	Test	p-value
Age			t = 1.09	0.91
Mean ± SD	35.22 ± 5.53	35.52 ± 5.9		
Median (minimum-maximum)	38 (20-50)	38 (20-50)		
Gender			X^2^ = 0.44	0.35
Male	15 (75%)	14 (70%)		
Female	5 (25%)	6 (30%)		
AO classification			X^2 ^=6	<0.001
43A1.1	2 (10%)	10 (50%)		
43A1.2	6 (30%)	4 (20%)		
43A1.3	2 (10%)	4 (20%)		
43A2.1	2 (10%)	0		
43A2.2	4 (20%)	0		
43A2.3	4 (20%)	2 (10%)		
Fibular fixation			X^2 ^= 4	0.04
Intact	2 (10%)	3 (15%)		
Fixed	10 (50%)	7 (35%)		
Not fixed	8 (40%)	10 (50%)		
Intraoperative blood loss (mL)			t =16	<0.001
Mean ± SD	91.1±25.2	222.1±70.1		
Median (minimum-maximum)	100 (70-120)	250 (150-300)		

Outcomes

In the MIPO group, the mean time to union was 17 ± 4 weeks, whereas in the IMN group it was 14 ± 2.5 weeks. There was a significant difference between the two groups, with the expert nail showing a shorter union time than MIPO. Regarding functional outcomes, in the MIPO group, 12 (60%) patients had excellent results, 6 (30%) good, and 2 (10%) fair, while in the IMN group, 14 (70%) were excellent, 4 (20%) good, and 2 (10%) fair. A significant difference was observed in the AOFAS scores, with the expert nail group showing superior results in both excellent and good categories. As for secondary procedures, in the MIPO group, 1 (5%) required revision and 3 (15%) required debridement, whereas in the IMN group, 2 (10%) underwent dynamization. No secondary procedures were needed in 16 (80%) MIPO cases and 18 (90%) IMN cases. The difference between groups regarding the need for secondary procedures was also statistically significant (Table 2).

**Table 2:** Comparative analysis of post-operative outcomes: time for union, AOFAS functional score, and need for secondary procedures in the MIPO and IMN MIPO: minimally invasive plate osteosynthesis, IMN: intramedullary nailing, SD: standard deviation, AOFAS: American Orthopaedic Foot and Ankle Society.

Variables	MIPO (n = 20)	IMN (n = 20)	Test	p-value
Time for union, weeks			t = 16.00	<0.001
Mean ± SD	17 ± 4	14 ± 2.5		
Median (minimum-maximum)	17 (12-24)	12 (12-20)		
AOFAS score			X^2 ^= 0.08	0.45
Excellent	12 (60%)	14 (70%)		
Good	6 (30%)	4 (20%)		
Fair	2 (10%)	2 (10%)		
Poor	0	0		
Need for secondary procedures				
Revision	1 (5%)	0	X^2 ^= 0.42	0.38
Debridement	3 (15%)	0		
Dynamization	0	2 (10%)		
No	16 (80%)	18 (90%)		

Case 1

**Figure 1:**
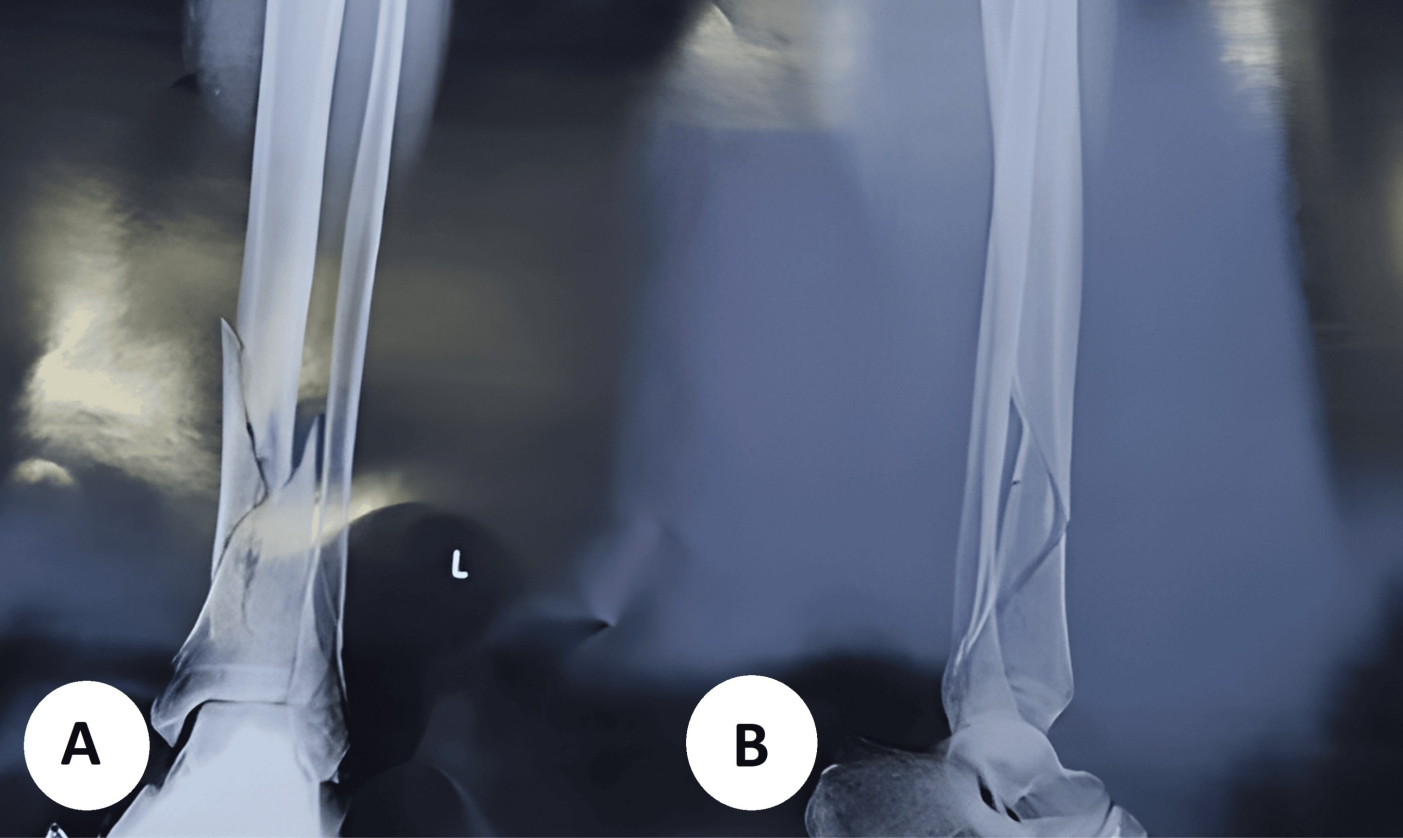
Preoperative radiographs of the left distal tibia (A) Anteroposterior view showing a displaced, extra-articular distal-third tibial shaft fracture with a long-oblique/spiral configuration and a small butterfly fragment; the ankle joint surface is preserved. (B) Lateral view confirming the oblique fracture plane with sagittal angulation and translation of the distal fragment, without evident intra-articular extension.

**Figure 2:**
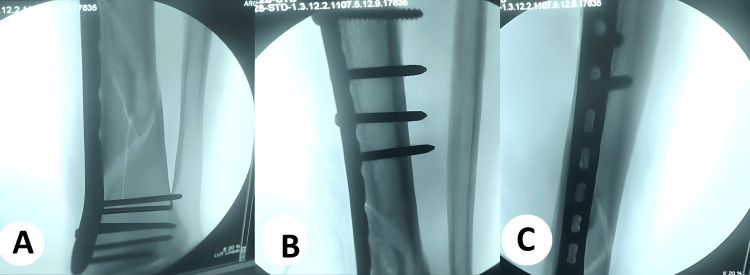
Intraoperative minimally invasive plate osteosynthesis (MIPO) technique for distal tibial fracture (A) Closed indirect reduction with submuscular tunneling; a pre-contoured distal tibial locking plate is slid in through a small anteromedial incision and secured first with distal locking screws, confirmed extra-articular on fluoroscopy. (B) The plate is advanced proximally and held to bone; proximal bicortical screws are inserted percutaneously through drill sleeves, maintaining a bridging construct without opening the fracture. (C) Final fluoroscopic check demonstrating restored coronal/sagittal alignment, appropriate screw lengths, and stable locked-plate fixation.

**Figure 3:**
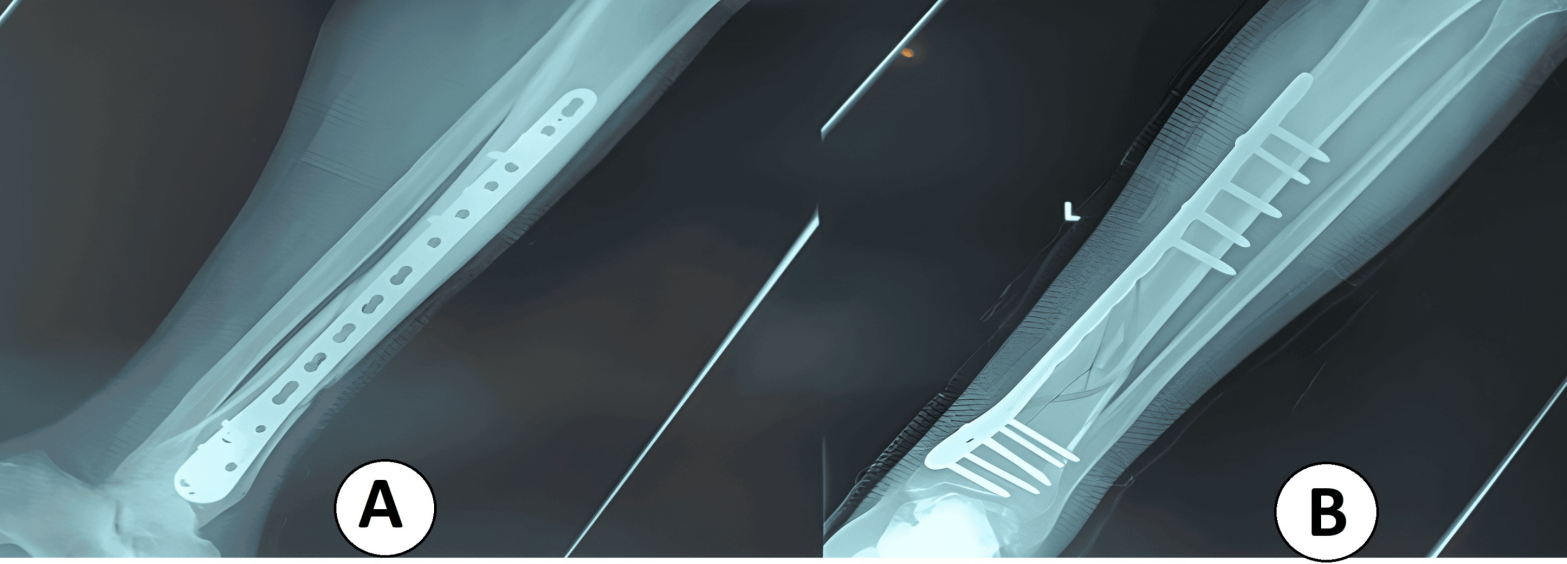
Immediate postoperative radiographs after MIPO locked plating of a distal tibial fracture (A) AP view: long bridging distal tibial plate spanning the metaphyseal–diaphyseal junction with clustered distal locking screws; alignment and length restored. (B) Lateral view: extra-articular plate position with appropriate screw lengths and maintained sagittal alignment; stable locked construct without joint penetration. MIPO: minimally invasive plate osteosynthesis.

**Figure 4:**
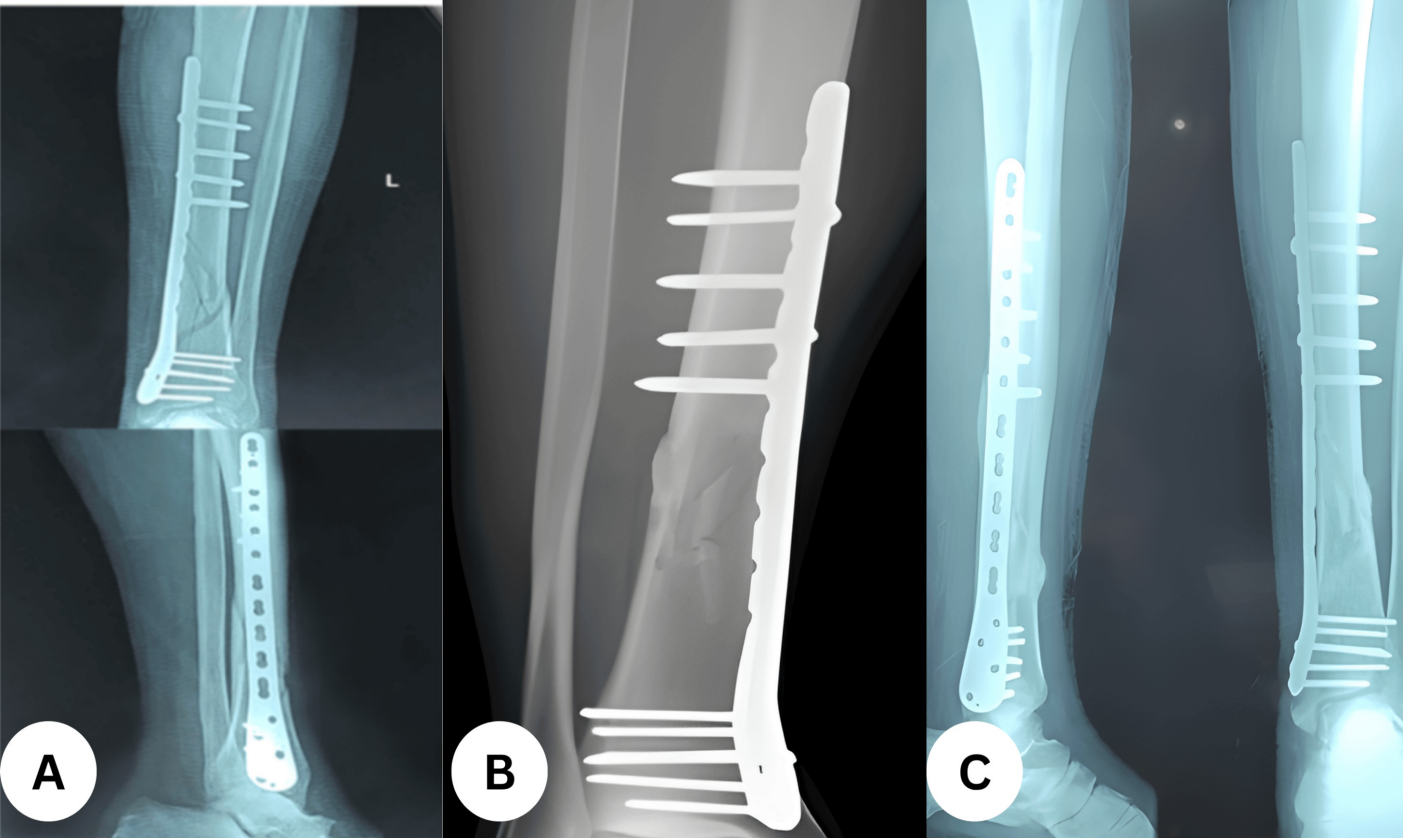
Serial follow-up after MIPO distal tibial locked plating (A) One-month radiograph showing maintained alignment with early callus formation; (B) three-month radiograph demonstrating progressive callus bridging; (C) six-month radiograph with cortical bridging consistent with union; hardware intact without loss of reduction. MIPO: minimally invasive plate osteosynthesis.

A 51-year-old male patient, a non-smoker, presented following a road traffic accident (RTA) with a left tibial fracture classified as AO 43A2 and Tscherne C1 (closed fracture) [17]. Under spinal anesthesia, closed reduction and internal fixation were performed using a distal tibial locked plate through the MIPPO technique. The total operative time was 116 minutes (Figures 1-4).

Case 2

**Figure 5:**
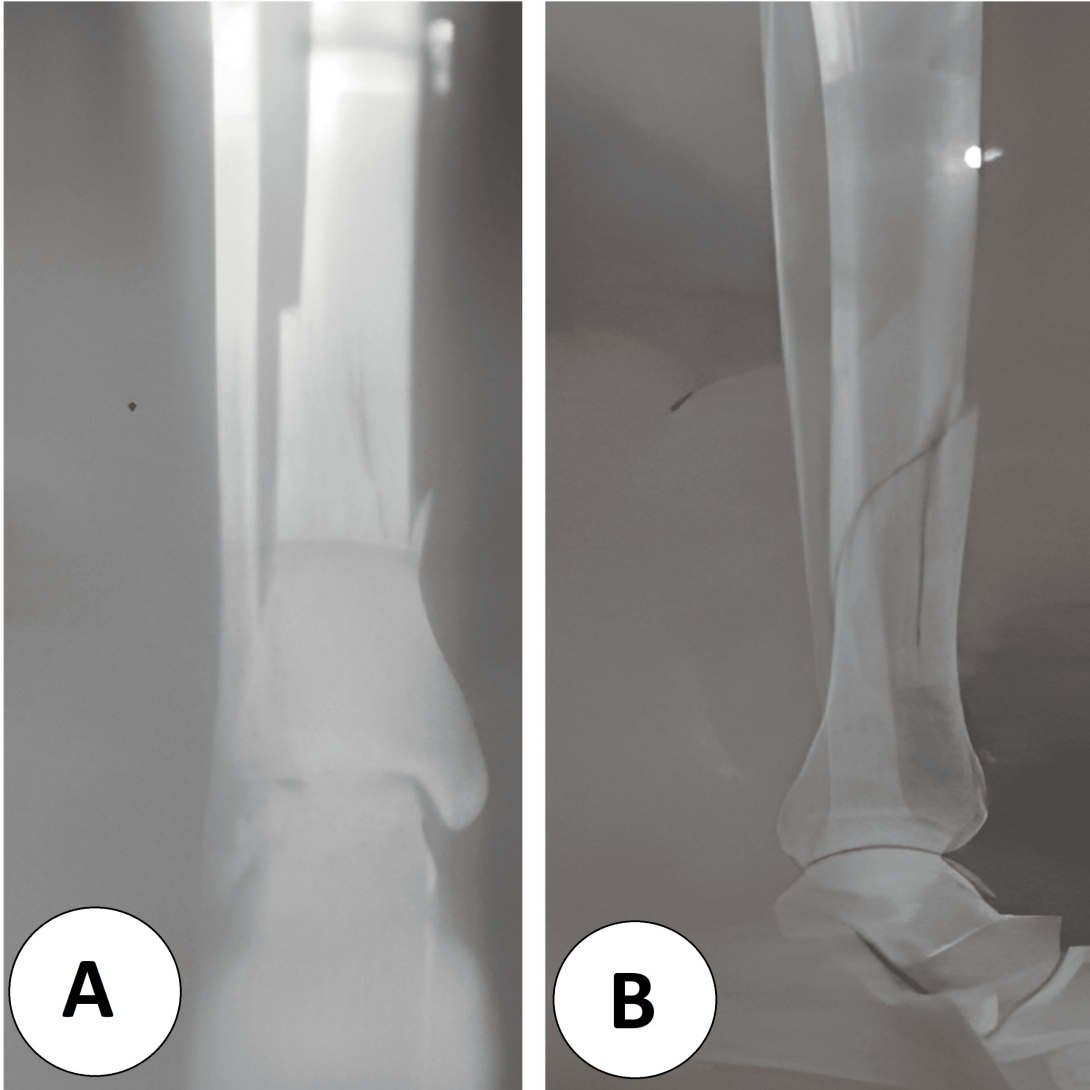
Preoperative injury radiographs (A) AP view showing an extra-articular distal-third tibial spiral/oblique fracture with metaphyseal comminution; ankle joint line preserved. (B) Lateral view demonstrating the long oblique fracture plane with a posterior spike and minimal displacement, without intra-articular extension.

**Figure 6:**
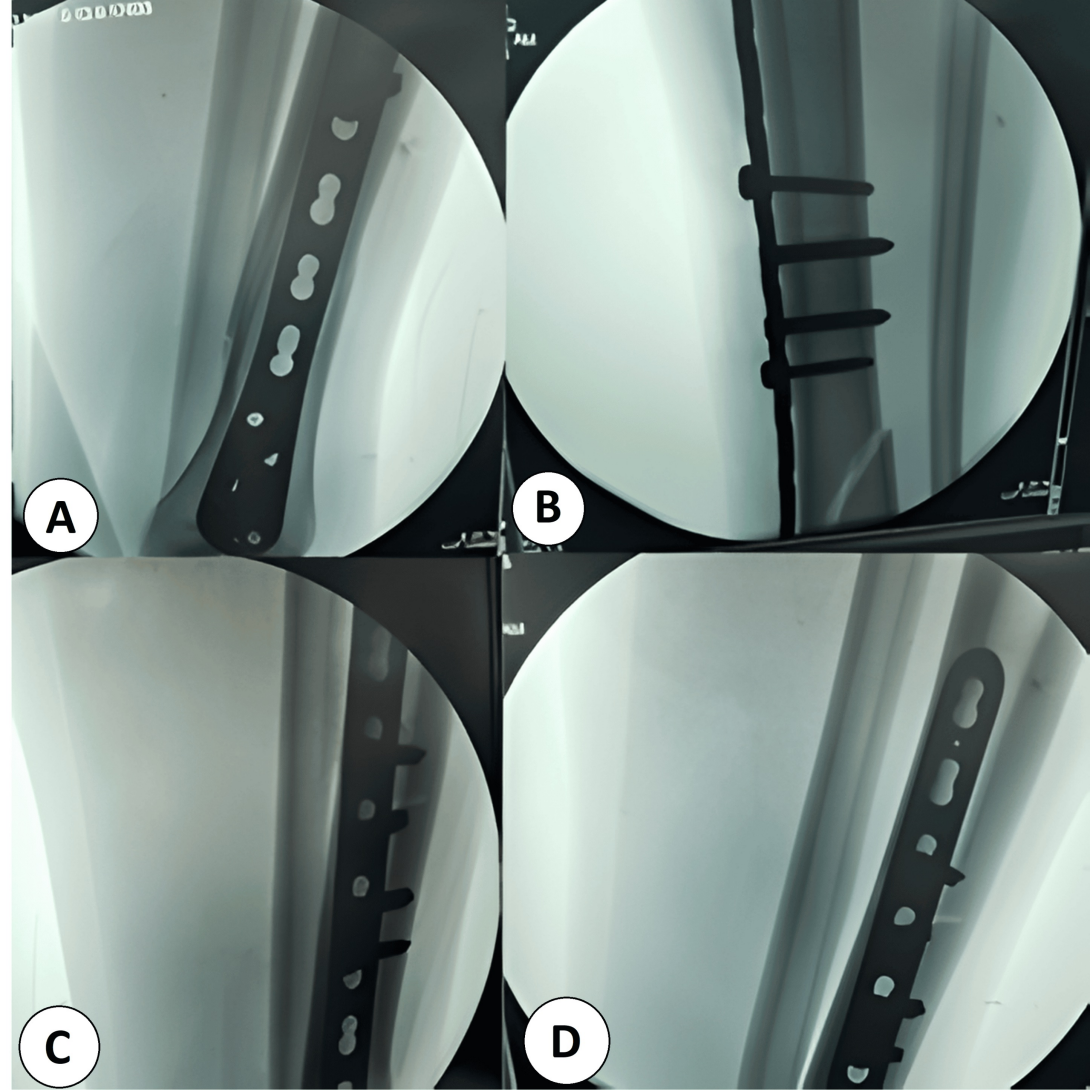
Intraoperative fluoroscopy during MIPO plating of a distal tibial fracture (A) Plate introduced submuscularly through a small anteromedial window and positioned extra-articular across the fracture. (B) Distal locking screws inserted to anchor the plate and maintain reduction. (C) Proximal percutaneous bicortical screws placed to create a bridging construct without opening the fracture site. (D) Final AP screening confirming restored length, axial/sagittal alignment, appropriate screw lengths, and stable fixation. MIPO: minimally invasive plate osteosynthesis.

**Figure 7:**
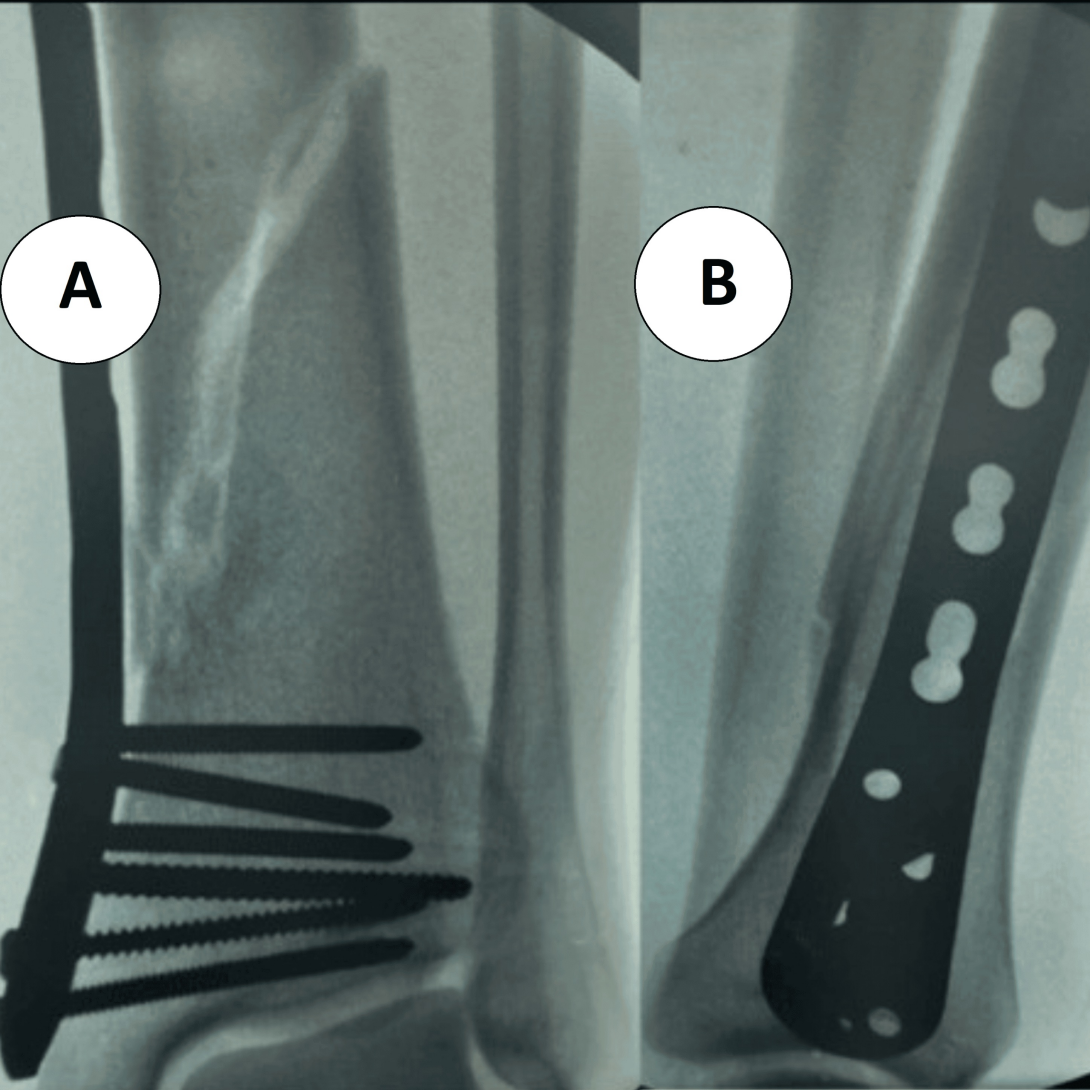
Postoperative follow-up after MIPO plating of a distal tibial fracture (A) AP ankle view showing clustered distal locking screws seated subchondrally with preserved ankle mortise and early callus along the medial cortex. (B) Lateral view confirming extra-articular plate position, appropriate screw lengths, maintained sagittal alignment, and progressive cortical bridging without hardware complications. MIPO: minimally invasive plate osteosynthesis.

**Figure 8:**
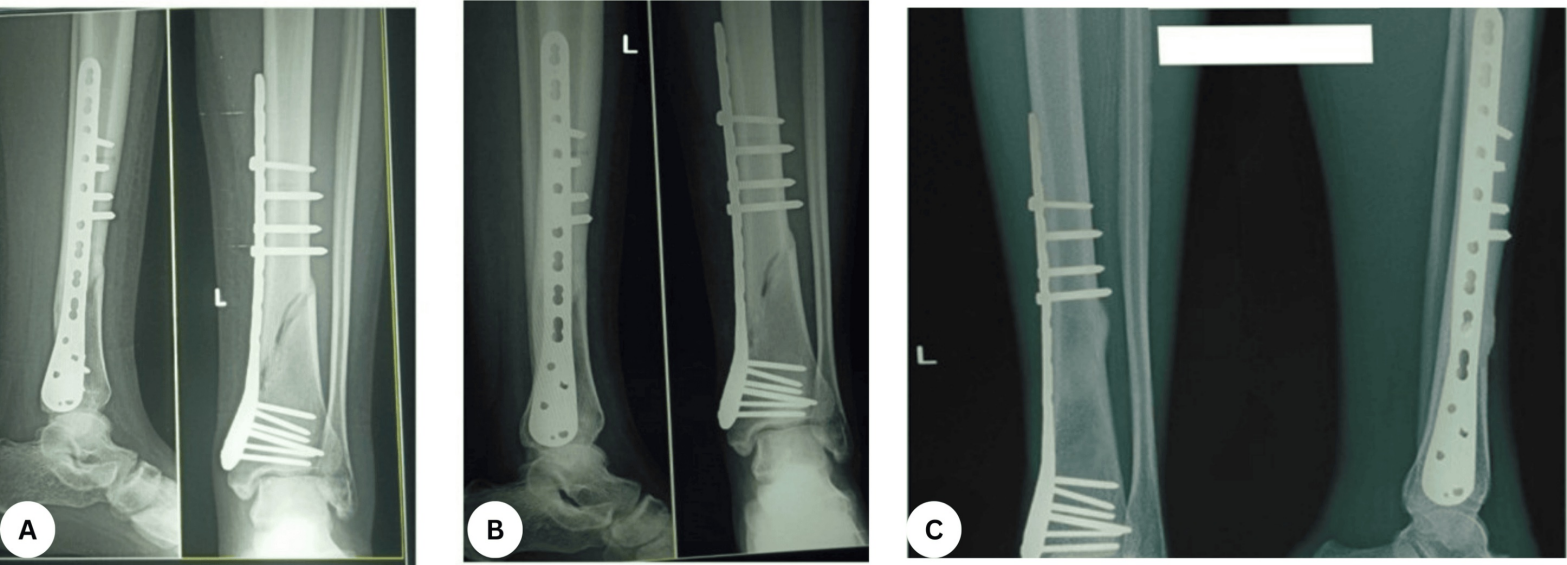
Serial postoperative radiographs after MIPO plating of a distal tibial fracture (AP and lateral views in each panel) (A) Early postoperative: plate spanning the metaphyseal–diaphyseal junction with clustered distal locking screws; alignment and length restored. (B) Intermediate follow-up: progressive callus with cortical bridging; hardware intact. (C) Late follow-up: circumferential cortical bridging consistent with solid union; no implant failure or loss of reduction. MIPO: minimally invasive plate osteosynthesis.

A 48-year-old male manual worker, a nonsmoker with no comorbidities, presented with a closed left tibial fracture (AO classification 43A2; Tscherne C2) following a fall to the ground. Under spinal anesthesia, closed reduction and fixation were performed using a distal tibial locked plate through the MIPPO technique. The total operative time was 130 minutes (Figures 5-8).

## Discussion

The present study revealed that, according to the time for union in the examined groups, in the MIPO group, the mean time for union was 17 ± 4. In the IMN group, the time for union was 14 ± 2.5. There were significant variances between the two groups regarding the time for union, and the expert nail had less time for union than MIPO. The statement about the significant difference in time to union highlights an important finding in the study: IMN appears to facilitate faster healing compared to MIPO. This result supports the hypothesis that IMN may be a better option for promoting quicker bone healing in lower third tibia shaft fractures, which can positively impact patient recovery times and overall outcomes.

The tibia is largely subcutaneous with minimal muscle coverage. Fractures of the distal third of the tibial shaft are among the most common injuries after high-energy trauma [18]. This segment has comparatively poor vascularity, which predisposes to delayed union. Treatment options include external fixation, intramedullary nailing, and plate fixation, all commonly employed [19].

Currently, MIPO and IMN are the principal approaches for extra-articular distal tibial fractures [20,21]. The optimal strategy remains contested [22]. Therefore, this study aimed to compare MIPO with distal tip-locking tibial nailing for lower third tibial shaft fractures, using both subjective and objective endpoints.

The present study revealed that, according to the time for union in the examined groups, in the MIPO group, the mean time for union was 17 ± 4. In the IMN group, the time for union was 14 ± 2.5. There were significant variances between the two groups regarding the time for union, and the expert nail had less time for union than MIPO. The significant difference in time to union highlights an important finding: IMN appears to facilitate faster healing compared to MIPO. This result supports the hypothesis that IMN may be a better option for promoting quicker bone healing in lower third tibia shaft fractures, which can positively impact patient recovery times and overall outcomes. This finding aligns with the study by Madhukar et al. [18], who aimed to compare the two most frequently applied methods, the tip-locking nail and MIPO, to detect an improved mode of fixation. Out of 42 cases with distal third tibial shaft fracture, separated into two groups of twenty-one cases in the MIPO group and twenty-one in the tip-locking nail group, they found that the mean union time was better with tip-locking nailing compared to plating.

Our outcomes are also consistent with Li et al. [19], who demonstrated that the union time was significantly higher in cases treated with a locked plate than in those treated with an expert nail (23.1 ± 3.6 vs 21.3 ± 3.5 weeks) with a p-value of 0.047. In addition, our outcomes are in concordance with Mohamed et al., who revealed that the mean interval for union in the expert nail group was 14 ± 2.85 weeks, whereas in the MIPO group it was 17.07 ± 4.01 weeks. This illustrates a statistically significant result in favor of the expert nail (p = 0.041), and the expert nail demonstrated a quicker duration to union. Another study by Köksal et al. found that the mean duration to union was significantly higher in the MIPO group (p = 0.001) [20,21].

In contrast, our findings disagreed with Zawam et al. [22], who reported that regarding the postoperative union time, the mean union time in the MIPO group was 16.73 ± 3.61 weeks versus 14 ± 2.8 weeks in the expert tibial nailing (ETN) group, with a p-value of 0.09, illustrating a nonstatistically significant variance between both groups.

Regarding the AOFAS score, in the MIPO group, 60% were excellent, 30% were good, and 10% were fair. In the IMN group, 70% were excellent, 20% were good, and 10% were fair. There were significant variances between the two groups regarding the AOFAS score, and results in the expert nail group were better than the MIPO group in excellent and good outcomes. The finding suggests that IMN leads to better functional recovery in terms of pain, mobility, and lower limb function. This result was significant because it helps demonstrate that although both techniques may offer positive results, IMN provides superior functional outcomes, making it a preferable option in cases where optimal functional recovery is critical.

In contrast, our findings disagreed with Zawam et al. [22], who reported that regarding the AOFAS score in group A, 40 (66.67%) patients were graded as excellent and 20 (33.33%) patients were good. In group B, 46 (76.67%) patients were graded as excellent, 10 (16.67%) patients were good, and four (6.67%) patients were fair (p = 0.78). The mean score of group A was 89.9%, and in group B was 90.26%.

In contrast to the current study, Mohamed et al. [21] reported that five cases in the MIPPO group had excellent AOFAS scores, four attained good scores, and one was classified as poor. In the expert nail group, seven cases achieved excellent outcomes, two cases attained good outcomes, and one case was fair. The p-value for the AOFAS score in the two groups was 0.392, indicating statistical insignificance.

Regarding alignment in the studied groups, we reported that in the MIPO group, 5% developed malalignment. In the IMN group, 15% developed malalignment. A significant variance was observed between both groups regarding malalignment, and the MIPO group developed less malalignment than the expert nail group. The lower rate of malalignment in the MIPO group in comparison with the IMN group suggests that MIPO may provide better alignment and stability in fractures, especially in more complex or distal fractures. This result is important because it highlights a potential disadvantage of IMN in terms of alignment, even though it remains a highly effective method for tibial fracture treatment.

Our findings align with those of Polat et al. [23], who aimed to compare intramedullary nailing with MIPO for the management of extra-articular distal fractures of the tibial shaft. Twenty-five consecutive cases having distal extra-articular fractures of the tibia were identified. It was discovered that rotational malalignment was more pronounced in the IMN group (p = 0.027).

Conversely, our results contradicted those of Wang et al. [24], who reported the presence of seventeen malalignments, encompassing both angular and rotational malalignment, with six (5.5%) in the MIPO group and eleven (7.1%) in the IMN group. Insignificant distinctions existed between the groups for angular and rotational malalignment.

In our study, we revealed that regarding infection, in the MIPO group, 20% had superficial infection and 80% had no infection. In the IMN group, 15% had superficial infection and 85% had no infection. There were significant variances between the two groups regarding infection, and the MIPO group had a higher infection rate than the expert nail group. The higher infection rate in the MIPO group likely results from the surgical approach, involving more soft tissue manipulation and potentially larger incisions, which increases exposure to bacteria and contaminants.

In concordance with the present study, Wang et al. [24] revealed that in total, 14 surgical site infections (SSIs) were found, 10 (9.1%) in the MIPO group and four (2.6%) in the IMN group. There was one deep surgical site infection and nine superficial surgical site infections in the MIPO group. There was one deep surgical site infection and three SSI infections in the IMN group. The superficial SSI infection rate in the MIPO group (8.2%) was greater compared to that in the IMN group (6/15, 1.9%) (p < 0.05).

Similarly, our study can be supported by Kc et al. [25], who revealed that with regard to superficial infection, the MIPPO group was significantly greater compared to the IMIL group (8% versus 4%) with p = 0.001. As well, our results are consistent with Mohamed et al. [21], who revealed that regarding infection, three cases in the MIPPO group developed deep infection, whereas in the expert nail group, two cases progressed to superficial infection, with the expert nail illustrating a reduced infection rate.

In contrast, our findings disagreed with Zawam et al. [22], who reported that in group A, eight (13.33%) patients sustained superficial infection versus six patients in group B (10%). A statistically insignificant variance was observed between the two groups.

In our study, we revealed that regarding malunion, in the MIPO group, 5% had nonunion and 10% had rotation. In the IMN group, 20% had coronal plane deformity. There were insignificant variances between the two groups regarding malunion. Along with our results, Polat et al. [23] reported that the rate of malunion was comparable among groups (p = 0.404).

Additionally, our findings align with those of Say et al. [26], who observed a statistically insignificant variance between the MIPO group and the IMN group regarding malunion (p = 0.412). In a meta-analysis, Guo et al. [27] found that the malunion rate was examined through a fixed-effects model, and they did not observe significant variances between the two groups (RR = 1.63, 95% CI 1.01 to 2.65, p = 0.05).

In their systematic review and meta-analysis, Liu et al. [28] demonstrated a significant distinction between the IMN group and the MIPO group, as indicated by the meta-analysis utilizing a fixed-effect model (RR = 1.85, 95% CI 1.21 to 2.83, p = 0.004), revealing a greater frequency of malunion in the IMN group compared to the MIPO group.

Also, in disagreement with the present study, Kc et al. [25] revealed that average malunion (degrees) in the MIPPO group was 5 (3-7) ± 1.41, whereas in the IMIL group, average malunion (degrees) was 10.22 (8 to 14) ± 2.04, which was statistically significant (p = 0.001).

The limitations of this study should be carefully considered. Methodologically, the prospective comparative design lacked a formal a priori sample size or power calculation. The sample size was instead determined by the number of eligible patients available during the recruitment period. This approach may have resulted in the study being underpowered to detect modest differences in key outcomes, such as time to union or AOFAS scores. Therefore, nonsignificant findings must be interpreted with caution. Allocation was nonrandom and at the surgeon's discretion; baseline differences, including AO/OTA classification and fibular fixation, indicate potential selection bias.

Further limitations include the six-month follow-up, which may not adequately capture late complications or long-term functional status. The study design is also constrained by the lack of blinding and the potential for selection bias, performance bias, and unaddressed learning-curve effects. Finally, the scope of outcomes was narrow, omitting a cost-effectiveness analysis and patient-reported outcome measures beyond the AOFAS score.

## Conclusions

IMN achieved a significantly shorter time to union and superior functional outcomes, as reflected by higher AOFAS scores, indicating faster recovery and better overall performance. Conversely, MIPO was associated with lower rates of malalignment, likely due to its enhanced control over fracture reduction, although it presented a higher infection rate compared to IMN. These findings suggest that IMN provides a more favorable option for achieving faster bone healing and improved function, while MIPO remains advantageous in preserving alignment, particularly in complex fracture configurations. Overall, both techniques are effective for distal tibial fractures, and the selection should be individualized based on fracture pattern, soft tissue condition, and surgeon expertise. Further studies with larger cohorts and longer follow-up are recommended to confirm and refine these outcomes.
